# Nerve Growth Factor Stimulates Cardiac Regeneration via Cardiomyocyte Proliferation in Experimental Heart Failure

**DOI:** 10.1371/journal.pone.0053210

**Published:** 2012-12-31

**Authors:** Nicholas T. Lam, Peter D. Currie, Graham J. Lieschke, Nadia A. Rosenthal, David M. Kaye

**Affiliations:** 1 Heart Failure Research Group, Baker IDI Heart and Diabetes Institute, Melbourne, Australia; 2 Department of Medicine, Alfred Hospital, Monash University, Melbourne, Australia; 3 Australian Regenerative Medicine Institute (ARMI), Monash University, Melbourne, Australia; University of Udine, Italy

## Abstract

Although the adult heart likely retains some regenerative capacity, heart failure (HF) typically remains a progressive disorder. We hypothesise that alterations in the local environment contribute to the failure of regeneration in HF. Previously we showed that nerve growth factor (NGF) is deficient in the failing heart and here we hypothesise that diminished NGF limits the cardiac regenerative response in HF. The capacity of NGF to augment cardiac regeneration was tested in a zebrafish model of HF. Cardiac injury with a HF phenotype was induced in zebrafish larvae at 72 hours post fertilization (hpf) by exposure to aristolochic acid (AA, 2.5 µM, 72–75 hpf). By 168 hpf, AA induced HF and death in 37.5% and 20.8% of larvae respectively (p<0.001). NGF mRNA expression was reduced by 42% (p<0.05). The addition of NGF (50 ng/ml) after exposure to AA reduced the incidence of HF by 50% (p<0.01) and death by 65% (p<0.01). Mechanistically, AA mediated HF was characterised by reduced cardiomyocyte proliferation as reflected by a 6.4 fold decrease in BrdU+ cardiomyocytes (p<0.01) together with features of apoptosis and loss of cardiomyocytes. Following AA exposure, NGF increased the abundance of BrdU+ cardiomyocytes in the heart by 4.8 fold (p<0.05), and this was accompanied by a concomitant significant increase in cardiomyocyte numbers. The proliferative effect of NGF on cardiomyocytes was not associated with an anti-apoptotic effect. Taken together the study suggests that NGF stimulates a regenerative response in the failing zebrafish heart, mediated by stimulation of cardiomyocyte proliferation.

## Introduction

Heart failure (HF) is one of the most prevalent forms of chronic cardiovascular disease. It accounts for a considerable proportion of death, disability and health care expenditure particularly in individuals over 65 years of age. Pathophysiologically, HF typically represents the end result of myocardial damage in association with cardiomyocyte loss [1] which contributes importantly to progressive ventricular remodelling. Unlike other organs such as the liver and bone marrow, the regenerative capacity of the myocardium is insufficient to mount a substantive regenerative response within the current clinical context [2]. However, with the recognition that a pool of cardiac progenitor cells exist in the heart [3] and the potential capacity of cardiomyocytes (CMs) to proliferate [4], [5], there has been considerable interest in the development of strategies for exploiting the possibility of cardiac regeneration in the prevention and treatment of HF [6].

Recently, the cardiac surgical resection model in zebrafish [7], [8], [9], [10], [11], [12] and neonatal mice [13] has been successfully exploited to study myocardial regeneration. These studies have demonstrated that in this experimental construct, there exists a regenerative potential within the heart, possibly arising from within the epicardium. Whilst these studies have provided novel insights into the cardiac response to acute injury, the relevance of these studies to HF is limited, as they do not recapitulate the progressive nature of HF. In particular, they also exclude the potential influence of important aspects of the pathophysiology of HF including the presence of cardiomyocyte apoptosis and alterations in the expression profile of neurohormones and cytokines which may modify a potential innate regenerative response.

Activation of the sympathetic nervous system is also a pivotal feature of progressive heart failure, and we previously showed that the magnitude of the activation of cardiac sympathetic nerves was strongly associated with the risk of death from heart failure [14]. In conjunction, a key component of the altered sympathetic nervous system pathology is a reduction in sympathetic nerve density, which we have demonstrated to be associated with a reduction in the tissue levels of nerve growth factor (NGF) both in experimental animals and humans [15]. NGF is a prototypic member of the neurotrophin family, and was initially recognized as a pro-survival and pro-differentiation factor for sensory and sympathetic neurons [16]. Acting via its key cognate receptor it has also recently been demonstrated to exhibit angiogenic activities and pro-survival actions in the setting of acute myocardial ischemic injury [17], [18]. In the present study, we primarily sought to determine whether NGF could rescue the failing heart and in so doing determine its mode of reparative actions.

## Materials and Methods

### Fish Lines, Aquaculture

Wildtype AB, *Tg(cmlc2:GFP)* and *Tg(cmlc2:DsRed2-nuc*) zebrafish were reared by standard methods, maintained at 28°C, at 14/10 hour light/dark cycles. In brief, *Tg(cmlc2:GFP)* zebrafish express GFP in the myocardium and *Tg(cmlc2:DsRed2-nuc*) express dsRed in the nuclei of cardiomyocytes. From paired matings, embryos were grown in ‘egg water’ (EW, distilled water supplemented with 0.06 g/L sea salt) and staged by timing in hours post fertilization (hpf). *Tg(cmlc2:DsRed2-nuc*) zebrafish were kindly provided by Geoffrey Burns (Cardiovascular Research Centre, MGH and Harvard Medical School, Boston, MA). All animal experiments were performed in strict accordance with the relevant laws and institutional guidelines of Monash University (approval number: 2009-02BC). All efforts were made to minimize suffering.

### Zebrafish Heart Failure Model

In the present study we employed a cardiotoxin-induced model of heart failure in zebrafish larvae at 72 hpf, a timepoint after which basic heart formation is complete [19]. Aristolochic acid (AA, Sigma, St. Louis, MO, USA) has previously been shown to be cardiotoxic and induces heart failure (HF) in zebrafish embryos and larvae [20]. Zebrafish larvae at 72 hpf were incubated in AA (2.5 µM) for 3 hours (72–75 hpf), conditions optimised to induce HF in approximately 50% of fish. AA was removed by 5 washes of egg water, and relevant interventions (NGF, K-252a; Sigma) were commenced thereafter (76 hpf onwards). Zebrafish were visually assessed at 80, 96, 120, 144 and 168 hpf for survival, heart rate, presence of pericardial oedema, reduced heart rate and cardiac morphology.

### Zebrafish Heart Isolation

To allow specific measurement of cardiac gene expression in zebrafish, hearts were isolated from tricaine anesthetized zebrafish based on protocol described previously [21]. In brief, hearts were isolated from *Tg(cmlc2:GFP)* zebrafish at 96 hpf by mechanical homogenization, nylon mesh filtration and, and finally with collection of GFP+ myocardial tissue using a fluorescence dissecting microscope.

### RNA Extraction and Real Time PCR

Total RNA was isolated from ∼150–200 zebrafish hearts per treatment group, per individual experiment, using TRIzol® (Invitrogen, Carlsbad, CA, USA), treated and purified (DNA-free kit, Ambion Applied Biosystems) based on the manufacturers’ instructions. RNA was quantified using the Nanodrop 2000 spectrophotometer (Thermo Scientific), and stored at −80°C. Messenger RNA (mRNA) was converted to cDNA by reverse transcription. Synthesis of first strand complementary DNA used Multiscribe Transcriptase. All reagents used in cDNA synthesis via the reverse transcription reaction were supplied from Applied Biosystems (Foster City, CA, USA) and based on its protocol. Quantitative Real time PCR was performed in triplicate using SYBR Green PCR Master mix (Applied Biosystems, Warrington, UK) based on the manufacturer’s instructions. Primers included: gata4 (5′-3′: fwd CACCGGGCACCATCATTC and rev CGGAGGACTGGTGGAGAAAG), caspase3 (5′-3′: fwd GACCGGCTCATGGTTCATTC and rev AGCTCCAGTTCACTGCCATACTT) and NGF (5′-3′: fwd GCCCGCCATTGGAACTC and rev TGAAGTCAGCGCACGTACAAA). Real time PCR was conducted on an Applied Biosystems 7300 Real Time PCR System, with 7300 System SDS (Sequence Detection System) Software (Applied Biosystems). Gene expression values were normalised to GAPDH housekeeping gene to obtain relative mRNA values. From the reactions, individual threshold cycle (Ct) values were generated from each well. Concordant results for 3 wells were averaged, and relative quantification fold changes in gene expression were analysed using the ΔΔCt method.

### Whole Mount Immunohistochemistry

Following relevant interventions, *Tg(cmlc2:GFP)* zebrafish were incubated in 5-bromo-2`-deoxyuridine (BrdU) (Sigma, St. Louis, MO, USA) at a concentration of 10 mM for a period of 24 hours (76–100 hpf). Zebrafish were subsequently euthanased with tricaine, and fixed with 4% paraformaldehyde (PFA) (Sigma, St. Louis, MO, USA) in PBS overnight at 4°C. Based on previously published methods [8], whole mount immunohistochemistry (IHC) was performed on the intact PFA preserved *Tg(cmlc2:*GFP*)* zebrafish, with use of fluorescent secondary antibodies and Hoechst 33342 (Molecular Probes).

### Confocal Microscopy and Image Analysis

Confocal imaging was performed with an upright Nikon D-Eclipse C1 laser scanning confocal microscope in association with Nikon NIS-Elements AR 3.2 64-bit software. Samples were captured using a 60X/1.0W DIC N2 ∞/0 WD 2.8 Nikon Japan NIR Apo water immersion objective. To generate 3D reconstructions of the entire heart, images were collected as a z stack at intervals of 1 µm for up to 130 µm depending on the heart sample thickness. To observe nuclei (Hoechst 33342), GFP [Tg(*cmlc2:*GFP)] and BrdU (Alexa Fluor 555), lasers at 408 nm, 488 nm, and 561 nm respectively were used. IMARIS x64 7.2.3 BITPLANE Scientific Software was used to analyse the confocal images. The total number of BrdU+ cardiomyocytes was determined from the z stack reconstruction of each heart in IMARIS, creating a mask of cardiomyocytes in the heart based on GFP and by identifying cells that demonstrated fluorescence for both GFP and BrdU. Cardiomyocyte nuclei labelled by DsRed2 in Tg(*cmlc2*:DsRed2-nuc) zebrafish were detected from z stack of the entire heart on the 561 nm laser. Total cardiomyocytes were counted from Tg(*cmlc2*:DsRed2-nuc) zebrafish using IMARIS.

#### TUNEL staining

For detection of apoptosis, Transferase dUTP Nick End Labeling (TUNEL) was conducted on 14 µm frozen sections from 100 hpf *Tg(cmlc2:DsRed2-nuc*) zebrafish embedded in OCT (optimal cutting temperature, Tissue-Tek, Sakura) using the In situ cell death detection kit, POD (Roche) based on the manufacturer’s protocol. Images were analysed on IMARIS to model TUNEL+ cells which co-localise with *Tg(cmlc2:DsRed2-nuc*) cardiomyocytes.

### Statistical Analysis

Group data are presented as mean ± standard error of the mean. Between-group comparisons were performed using an unpaired students t-test or ANOVA as appropriate. Kaplan-Meier survival curves were constructed to evaluate the mortality and heart failure incidence, and between group comparisons were performed using the Mantel-Cox log rank test. A p value of <0.05 (p<0.05) was considered statistically significant. Statistical tests were conducted using a commercially available software package (SPSS Statistics 17.0).

## Results

### Morphologic and Molecular Profile of Zebrafish Heart Failure

**Figure 1:**
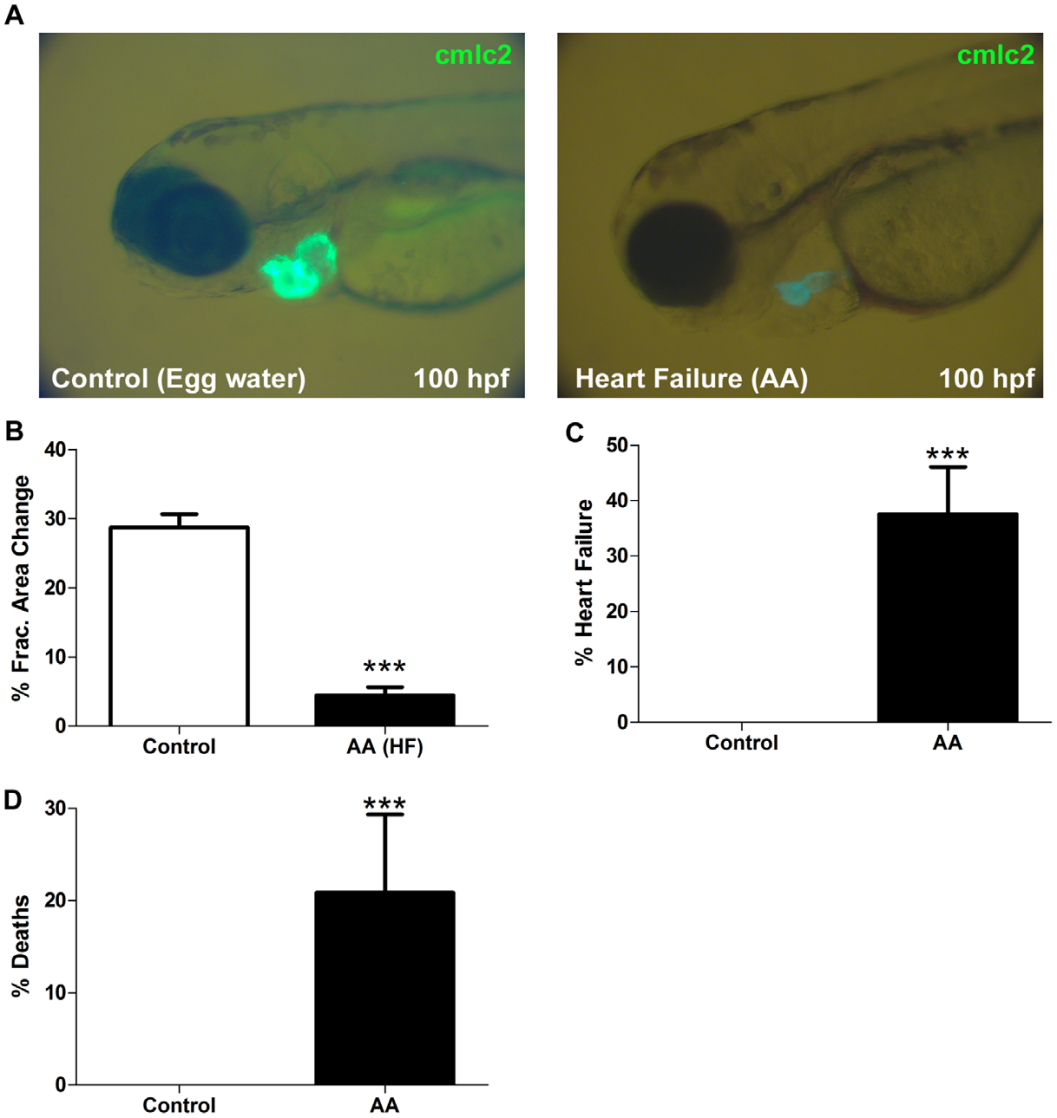
Experimental heart failure model in zebrafish. **A** Zebrafish (100 hpf) without HF (left panel) and with the HF phenotype (right panel). Bar graphs showing **B.** Ventricular fractional area change at 96 hpf, **C.** Incidence of AA induced HF at 168 hpf. **D.** Incidence of AA induced mortality at 168 hpf. ***p<0.001.

We first characterised the concentration and time dependent cardiotoxic actions of AA in zebrafish. We found that there was a temporal and dose related (data not shown) effect of AA in inducing a heart failure phenotype marked by impaired altered cardiac morphology, pericardial oedema, and reduced contractility (Figure 1A and 1B). Arising from these preliminary studies we selected an AA exposure concentration of 2.5 µM for 3 hours duration from 72–75 hpf. Using this regimen, the HF phenotype developed in 37.5% of larvae exposed to AA by 168 hpf (p<0.001, n = 96), compared to controls in which no HF developed (Figure 1C). As shown in Figure 1D, AA exposure and HF development was associated with a mortality rate of 20.8% by 168 hpf, compared with controls where none died (p<0.001, n = 96).

**Figure 2:**
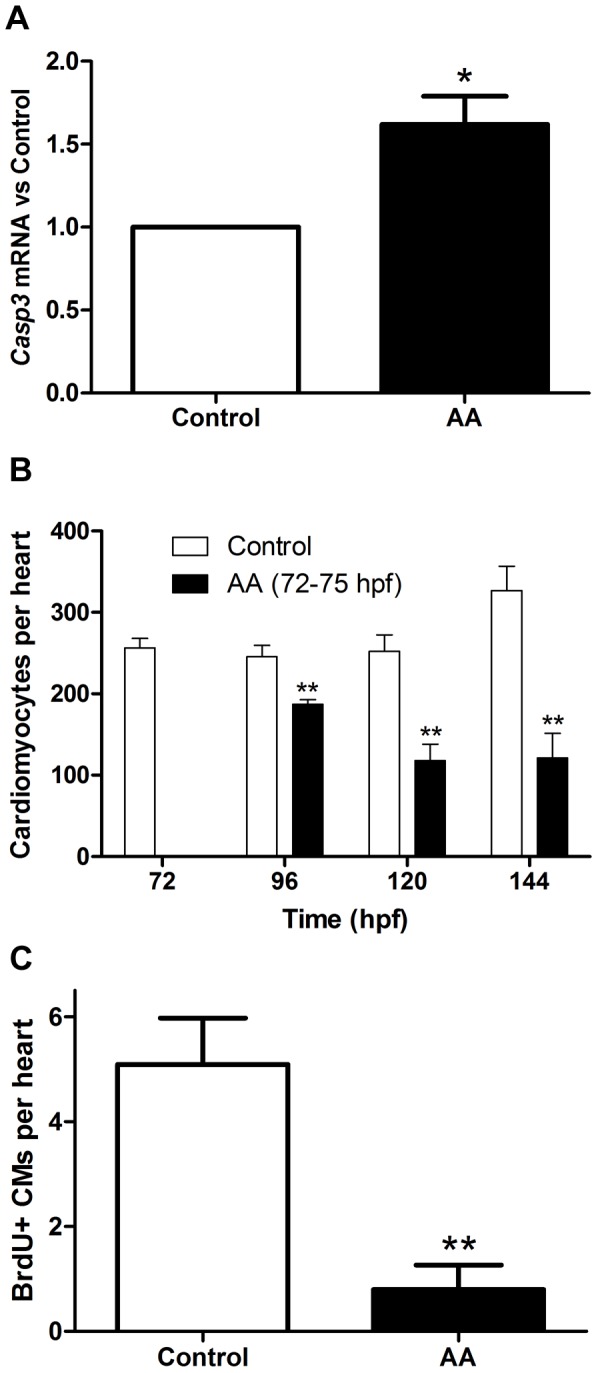
Aristolochic acid increases apoptosis in the heart, deplete CMs and reduces CM proliferation. Bar graphs demonstrating **A.** Expression of caspase 3 mRNA in the heart at 96 hpf, **B.** Time dependent changes cardiomyocyte number and **C.** Abundance of BrdU positive cardiomyocytes at 100 hpf.

In conjunction with the functional and morphologic assessment of this model of cardiotoxin mediated heart failure, we evaluated the cellular and molecular signature of this model of HF. Following exposure to AA (72–75 hpf), at 96 hpf there was a 62% increase (p<0.05, n = 6) in expression of caspase 3 mRNA in the heart (Figure 2A). This was accompanied by a progressive reduction in the total number of cardiomyocytes (p<0.01, n = 5, Figure 2B) and there was a significant reduction in the frequency of cardiomyocytes undergoing cell cycling as assessed by BrdU incorporation for 76–100 hpf (Figure 2C).

### NGF Rescues Zebrafish Heart Failure

**Figure 3:**
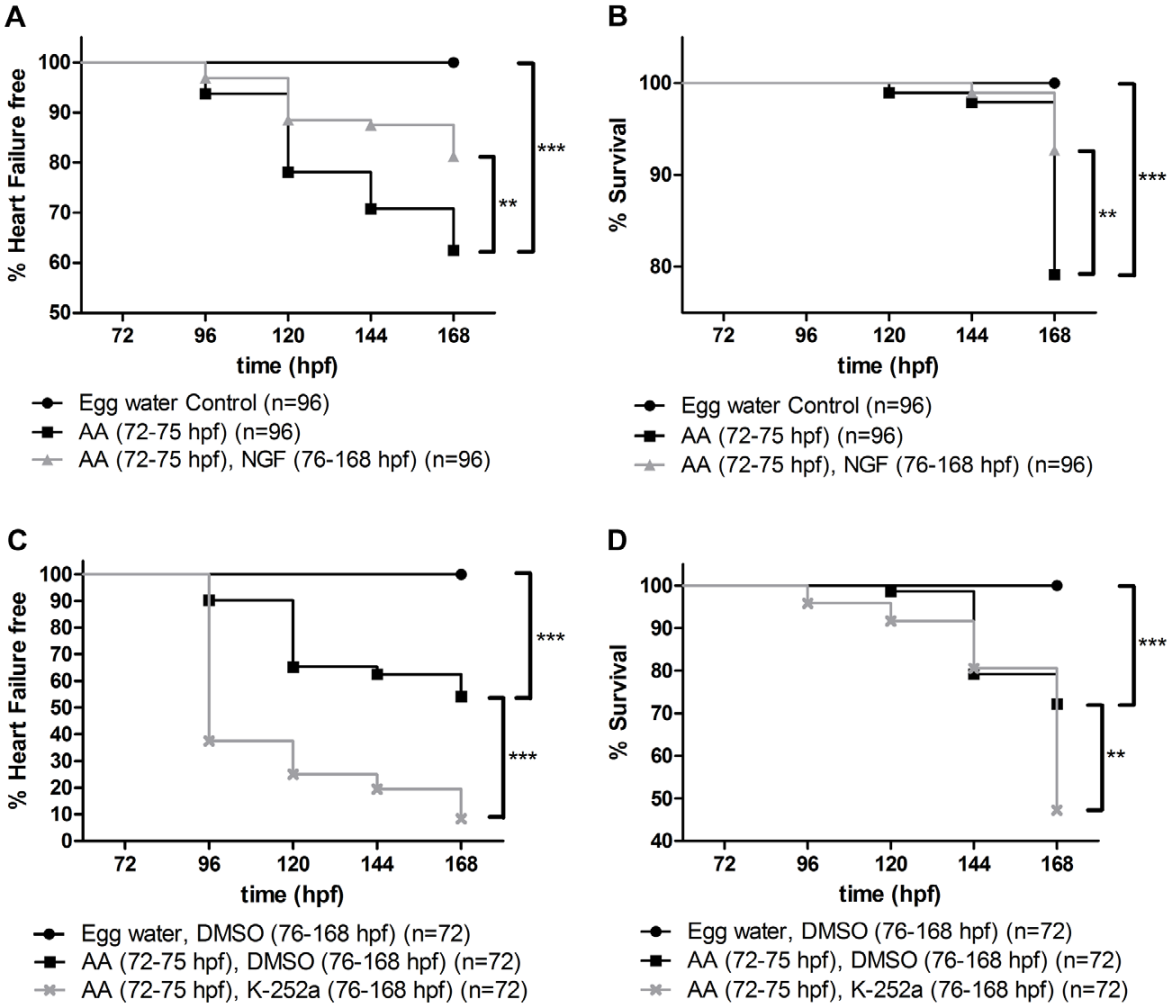
NGF decreases the incidence of AA induced HF and death. Kaplan Meier Curves showing **A.** Effect of NGF on frequency of HF, **B.** Effect of NGF on frequency of death and **C.** Effect of the NGF trkA receptor antagonist, K-252a, on frequency of HF. **D.** Effect of K-252a on frequency of death. **p<0.01, ***p<0.001.

To support our contention that reduced tissue NGF levels contribute to the pathogenesis of the response to cardiac injury and heart failure we evaluated the levels of NGF mRNA expression in AA treated zebrafish hearts. By RT-PCR we demonstrated a 42±13% reduction in NGF mRNA in AA treated fish (n = 6 per group, p<0.05). Next, in order to determine whether NGF could prevent or reverse the development of AA induced HF we treated zebrafish at 76 hpf with NGF (50 ng/ml) following removal of AA (72–75 hpf). As shown in Figure 3A, by 168 hpf NGF reduced the incidence of AA induced HF by 50% (p<0.01) and death by 65% (p<0.01; Figure 3B). Zebrafish larvae in egg water supplemented with NGF at this concentration did not have any visible effect on HF or death compared to egg water controls (data not shown).

To corroborate the protective actions of NGF shown above, we next investigated the effect of inhibiting the high affinity NGF (TrkA) receptor. In this series of experiments, after AA treatment and removal (72–75 hpf), zebrafish (76 hpf) were exposed to K-252a (100 nM), a serine/threonine protein kinase inhibitor that inhibits the high affinity NGF (trkA) receptor. TrkA receptor antagonism increased the incidence of HF by 1.9 fold (p<0.001) from 46% to 92% (Figure 3C), and of death (p<0.01) from 28% to 53% at 168 hpf (Figure 3D).

Continuous exposure of zebrafish to vehicle alone (0.01%DMSO) from 76 hpf following AA treatment (Figure 3C and 3D) and zebrafish continuously exposed to egg water supplemented with K-252a (76–168 hpf) at this concentration (data not shown) did not cause HF or death by 168 hpf.

### NGF Induces Cardiac Regeneration

**Figure 4:**
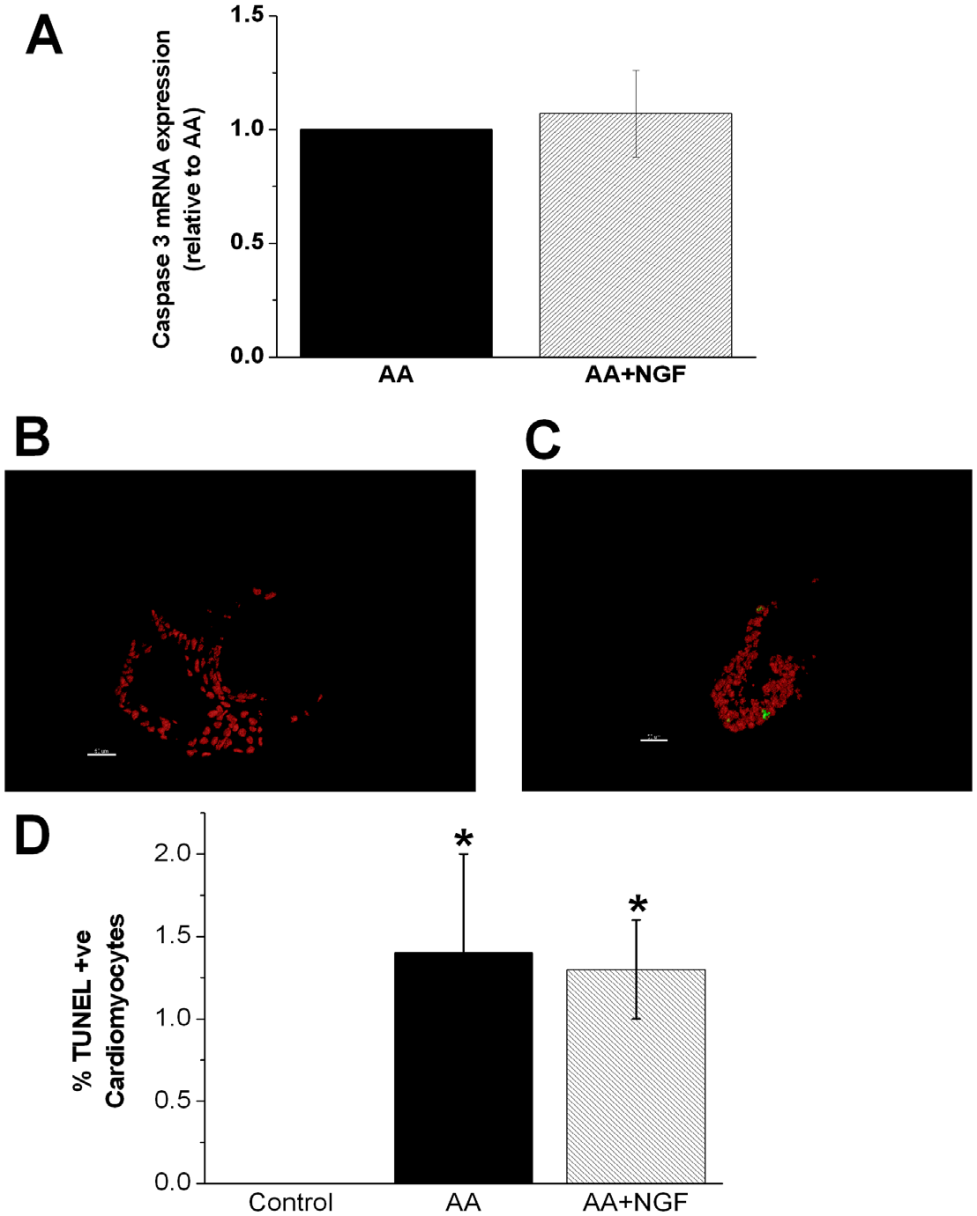
NGF does not attenuate AA induced apoptosis. A. Bar graph represents absence of effect of NGF on caspase 3 mRNA expression in AA treated (72–75 hpf) zebrafish hearts at 96 hpf. **B and C.** Representative IMARIS images of cardiomyocyte TUNEL staining in control and AA treated zebrafish heart at 100 hpf. Scale bar = 50 µm. **D.** Bar graph showing quantitative analysis of TUNEL staining (n = 6 per group, * p<0.05 vs control).

**Figure 5:**
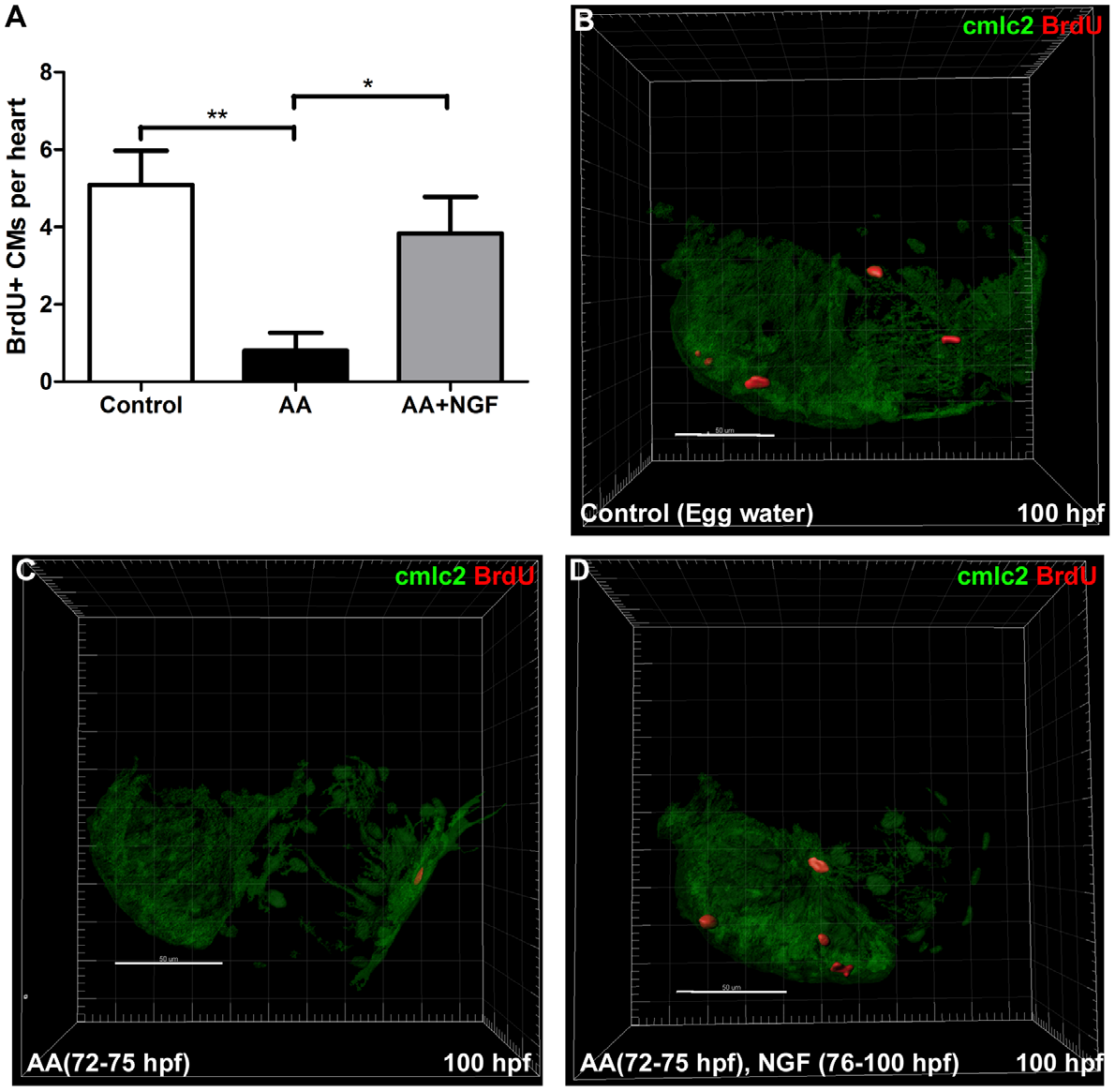
NGF stimulates CM proliferation following AA exposure. A. Bar graph showing the effect of NGF on cardiomyocyte proliferation. **B–D.** Representative IMARIS images of BrdU+ cardiomyocytes in the heart (76–100 hpf) from control (B), AA treated (C) and AA treated and post treated with NGF (D). Scale Bars  = 50 µm. *p<0.05, **p<0.01.

To determine the mechanism by which NGF prevented the development of heart failure, we considered the dual possibilities that NGF exerted an anti-apoptotic effect or facilitated cardiac regeneration. In isolated hearts at 96 hpf, the previously documented AA induced increase in caspase-3 mRNA expression (Figure 2A) was unaltered by the addition of NGF (Figure 4A), and remaining significantly greater than control (p<0.05). This was supported by terminal deoxynucleotidyl transferase dUTP nick end labeling (TUNEL) staining of 100 hpf heart sections from *Tg(cmlc2:DsRed2-nuc*) zebrafish which demonstrated a similar pattern. Specifically, quantitative evaluation of TUNEL staining showed that no TUNEL positive cardiomyocytes were detected in control hearts (Figure 4B), whilst in AA treated fish 1.4±0.6% of cardiomyocytes were TUNEL+ (p<0.05, n = 6 per group, Figure 4C) and this was unaltered in NGF treated AA exposed fish (1.3±0.3%, p<0.05 vs control; p = ns vs AA by ANOVA) as shown in Figure 4D. To determine whether NGF attenuated the development of HF via stimulation of cardiac regeneration due to cardiomyocyte proliferation, we determined the rate of BrdU incorporation in the first 24 hour period after AA was removed (76–100 hpf). As shown in Figure 5A, under basal conditions there were 5.1±0.9 BrdU+ cardiomyocytes per heart (CMs/heart) which fell to 0.8±0.5 BrdU+ CMs/heart (p<0.01, n = 10) following AA treatment (Figure 5B). After AA exposure, NGF treatment increased the number of BrdU+ CMs by 4.8 fold to 3.8±0.9 BrdU+ CMs/heart (p<0.05, n = 12) (Figure 5C).

**Figure 6:**
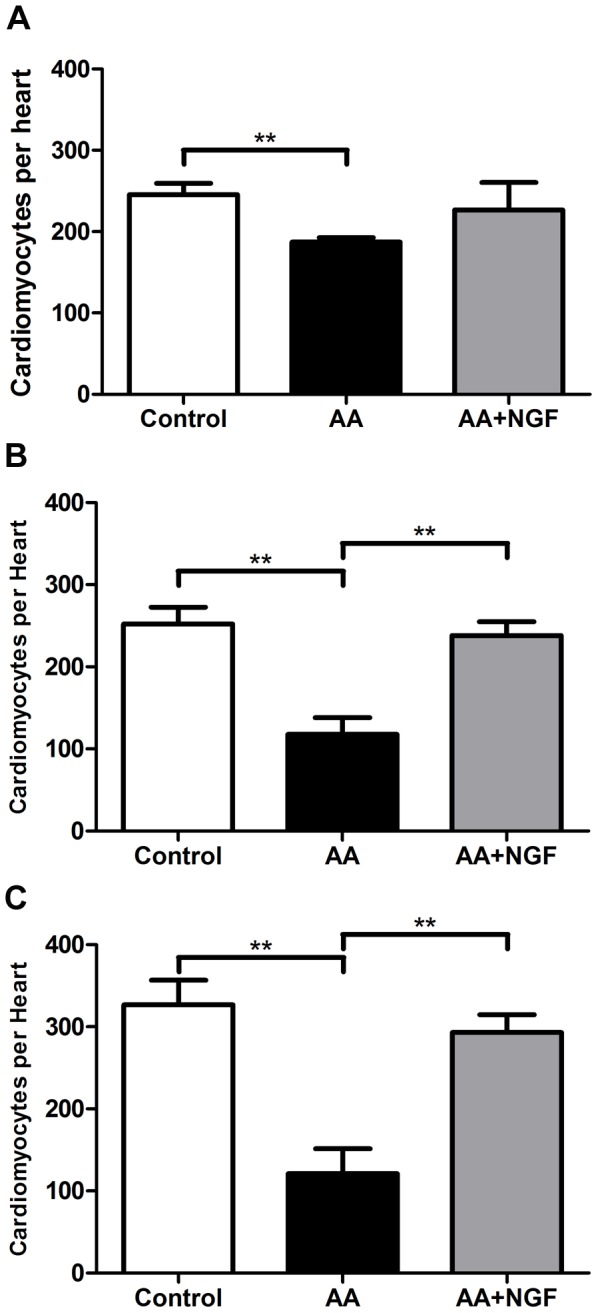
NGF restores total CM number following AA exposure. Bar graphs showing that NGF restores total cardiomyocyte number in zebrafish model of experimental heart failure. Time dependent effects at **A.** 96 hpf, **B.** 120 hpf, and **C.** 144 hpf. **p<0.01.

In order to assess whether the increased rate of BrdU incorporation was accompanied by an increase in the total number of cardiomyocytes in the heart, we used *Tg(cmlc2:DsRed2-nuc*) zebrafish which express DsRed2 fluorescent protein in the nuclei of cardiomyocytes [22], [23]. As evaluated by 2-way ANOVA, NGF significantly increased the time-dependent changes in cardiomyocyte numbers following AA exposure from 96–144 hpf (p<0.001, n = 15). More specifically, under basal conditions at 72 hpf, there were 256±12 (n = 5) cardiomyocytes per heart. Following AA exposure (72–75 hpf), at 96 hpf there was a 24% decrease in cardiomyocyte numbers to 187±6 (AA) per heart as compared to time-matched controls (p<0.01, n = 5; Figure 6A). In fish treated with AA subsequently exposed to NGF there was no significant evidence of regeneration at 96 hpf. By 120 hpf, the number of cardiomyocytes in the hearts of AA exposed zebrafish had decreased by 53% (118±21 vs 252±20 CMs/heart, p<0.01, n = 5; Figure 6B). By contrast, fish treated with AA and supplemented with NGF had a 102% greater number of cardiomyocytes (238±17 CMs/heart, p<0.01, n = 5; Figure 6B). As shown in Figure 6C, at 144 hpf AA treatment caused a 63% reduction in total cardiomyocyte numbers compared to controls (AA vs control: 121±30 vs 327±30 CMs/heart p<0.01, n = 5). The addition of NGF after AA treatment rescued cardiomyocyte cell numbers with a 142.3% increase to 293±22 CMs/heart (p<0.01, n = 5).

We next characterised the pattern of gene expression that accompanied the apparent regenerative actions of NGF in the zebrafish heart. On the basis of recent studies [10] we determined the level of expression of GATA4 and NKx2.5 mRNA within the zebrafish heart. In this model of HF, neither GATA4 nor Nkx 2.5 expression changed during the development of HF, and their expression was unaltered by the addition of NGF (data not shown).

**Figure 7:**
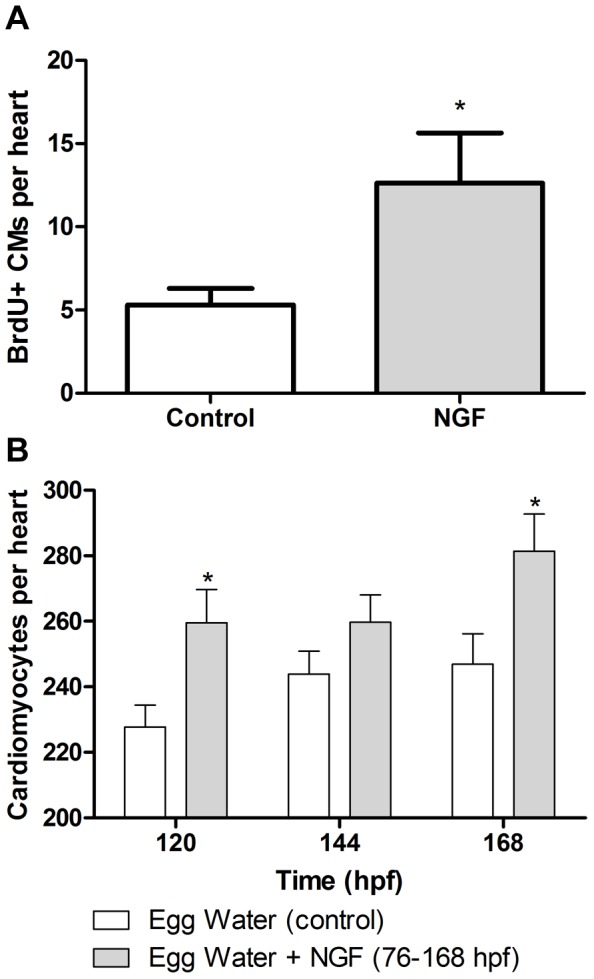
NGF induced CM proliferation and total CMs. Bar graphs show that fish exposed to egg water supplemented with NGF from 76 hpf onwards (grey bars) increases cardiomyocyte proliferation (**A**), and increases total cardiomyocyte number over time (**B**). *p<0.05.

To determine whether NGF causes cardiomyocyte proliferation, wholemount immunohistochemistry was conducted on *Tg(cmlc2:GFP)* zebrafish exposed to control conditions (Egg water) or NGF (50 ng/ml) in the presence of BrdU from 76–100 hpf. *Tg(cmlc2:GFP)* zebrafish were exposed to NGF (50 ng/ml) continuously in Egg water from 76 hpf, a timepoint from the AA model from which NGF was added after AA was removed. Addition of NGF caused a 2.38 fold increase in BrdU+ cardiomyocytes (control vs NGF: 5.3±1.0 vs 12.6±3.0 BrdU+ CMs/heart, p<0.05, n = 8–10 per group; Figure 7A). To assess the relationship between increased numbers of BrdU+ cardiomyocytes in NGF treated fish and total cardiomyocyte numbers, *Tg(cmlc2:DsRed2-nuc*) zebrafish were exposed to NGF (50 ng/ml) continuously in Egg water from 76 hpf. The number of cardiomyocytes in the heart were counted from confocal microscopy z-stacks of *Tg(cmlc2:DsRed2-nuc*) zebrafish on IMARIS. As determined by 2-way ANOVA, zebrafish exposed to NGF caused a significant increase in cardiomyocyte numbers per heart compared to control fish raised in egg water from 96–168 hpf (p<0.05, n = 37–39). In detail, as shown in Figure 7B, by 120 hpf NGF induced a 14% increase in total cardiomyocytes (p<0.05, n = 9) from 227.7±6.7 CMs/heart (control) to 259.6±10.2 CMs/heart (NGF). At 168 hpf, NGF continued to be associated with a 14% increase in total cardiomyocytes compared to fish from egg water controls (control vs NGF: 257±9.3 to 281.4±11.3 CMs/heart, p<0.05, n = 9–10, Figure 7B).

## Discussion

The present study demonstrates, for the first time, the ability of NGF to repair the heart by inducing cardiomyocyte proliferation in an experimental model of heart failure in larval zebrafish. In contrast to the widely used surgical resection model of cardiac regeneration in adult zebrafish [7], [9], [10], our model was designed to recapitulate aspects of the clinical phenotype of heart failure. In particular our model demonstrated a morphologic and outcome profile consistent with that of advanced HF. Furthermore, similar to clinical and experimental models of progressive HF the present cardiotoxic HF model features activation of a pro-apoptotic cascade.

In the previous zebrafish resection studies [7], [8], [9], [10], [11], it has been clearly demonstrated that an innate capacity for cardiac regeneration exists. Moreover emerging evidence suggests that this response may be mediated via the epicardium, perhaps triggered in part by the surgical process itself. In the present study we did not observe an automatic activation of intrinsic repair, as reflected by marked reduction in BrdU incorporation after cardiotoxin exposure together with a reduction in total cardiomyocyte number and by the absence of a GATA4 response. Our study is consistent with a previous study which suggest that a stimuli which results in the rapid loss of more than 60% of the cardiomyocytes may represent a point at which intrinsic repair is insufficient to recover [24]. In contrast to adult zebrafish with heart failure induced by genetic cardiomyocyte ablation, recovery was possible over a longer period [24]. Furthermore, in our model using *Tg(fli1:GFP*) zebrafish, AA caused a loss of endocardium (data not shown) consistent with the original AA HF model [20]. In addition we report that AA also causes a significant loss of cardiomyocytes which was not previously identified [20], and is an important factor in the progression of HF. Taken together, AA induced heart failure is more severe than the cardiomyocyte genetic ablation model [24] because it caused more than 60% cardiomyocyte loss in addition to significant loss of endocardium.

Our study was designed to specifically test the hypothesis that regenerative responses within the heart might be influenced by the altered relative tissue levels of neurohormones, cytokines and growth factors that could alter the activity of reparative mechanisms. Specifically, we have previously shown that marked alterations occur in the activity of the cardiac sympathetic nervous system in heart failure [14], together with a substantial depletion of nerve growth factor in the heart [15].

While NGF has been demonstrated to exert an anti-apoptotic effect in cardiomyocytes under conditions of ischemic damage [18], we did not observe an anti-apoptotic action in this study using the same concentration of NGF. Specifically, although caspase 3 mRNA levels and TUNEL positivity increased in the setting of experimental HF, this was unaffected by the subsequent treatment with NGF. While we did show an increase in cardiac caspase expression, we cannot specifically localize the expression to cardiomyocytes although few non-myocytes were evident. Beyond actions on cardiomyocytes per se, it has also been shown that NGF may exert beneficial actions via vascular effects, including the stimulation of angiogenesis following myocardial infarction [17].

In the present study we found that in zebrafish exposed to NGF there was an increase in BrdU+ cardiomyocytes and total cardiomyocytes, suggesting that NGF increases cardiomyocyte proliferation in vivo. This observation was completely consistent with our demonstration that the cardiotoxin induced reduction in cardiomyocyte numbers could be reversed by NGF. In this context it is of note that in chick dorsal root ganglion sensory neuronal cultures, NGF potently increased neuregulin-1 (NRG1) [25], and that NRG1 through its receptor ErbB4 induced mammalian adult cardiomyocytes to proliferate [5]. Together it is therefore possible that NGF may trigger proliferation of cardiomyocytes via upregulation of NRG1 in the heart. Recent work suggested that GATA4 is activated in cardiomyocytes during the regenerative process of the cardiac resection model [10], however in this study the addition of NGF following AA treatment did not change gata4 mRNA transcript levels in the heart.

Additionally, a recent study in adult mouse heart demonstrated that cardiomyocyte specific deletion of glycogen synthase kinase (GSK)-3β following surgically induced myocardial infarction increased cardiomyocyte proliferation [26]. Of interest, in rat sympathetic neurons, PC12 cells, and mouse embryonic dorsal root ganglion neurons, NGF promotes axon growth by activation of phosphatidylinositol 3-kinase (PI3K) which inactivate GSK-3β through the TrkA receptor [27], [28], [29], [30], [31]. In contrast, in mouse embryonic hippocampal neurons, NGF promotes axon elongation by inactivation of GSK-3β mediated by p75^NTR^ receptor [32]. Importantly, NGF ultimately inactivates GSK-3β regardless of which NGF receptor signalling pathway it activates. Taken together, it is conceivable that NGF may inactivate GSK-3β in cardiomyocytes to induce proliferation.

In adult zebrafish, cardiac regeneration is primarily mediated by cardiomyocyte proliferation [10], [11], and NGF is modestly upregulated in the heart post amputation [33]. Our study supports that NGF augments cardiomyocyte proliferation and may play an important role in cardiac regeneration. While our findings are consistent broadly with the concept of a cardiomyocyte proliferation mediated regenerative response, it also highlights the importance of differences in experimental paradigms. In the present study, in our globally injured heart model we show that there is an insufficient regenerative response, unlike that in the locally, surgically damaged zebrafish heart.

In summary, the present study demonstrates that NGF may attenuate the progression of HF via the induction of a regenerative program based upon cardiomyocyte proliferation rather than by an anti-apoptotic mechanism. These studies are complimentary to prior observations which demonstrate that NGF expression is reduced in the failing heart, and importantly taken together suggest that a deficiency of NGF within the failing heart could impair the capacity of any residual regenerative capacity. Importantly, further work is required to establish the translational relevance of these findings to that in human disease in older individuals. Regenerating the myocardium following injury to prevent the onset of heart failure is one of the ultimate goals of cardiac repair [6]. We hypothesise that NGF may provide a more favourable environment which sufficiently regenerates the heart to prevent the development of heart failure. Further studies are required to evaluate the precise molecular mechanism by which this process is mediated, and importantly to establish whether similar pathways exist in the adult mammalian heart.
